# To Catch a Virus, 2nd Edition

**DOI:** 10.3201/eid3002.231576

**Published:** 2024-02

**Authors:** Richard N. Danila

**Affiliations:** Retired, St. Paul, Minnesota, USA

**Keywords:** virology, history, diagnostics, COVID-19, respiratory infections, severe acute respiratory syndrome coronavirus 2, SARS-CoV-2, SARS, coronavirus disease, zoonoses, viruses, coronavirus, HIV/AIDS and other retroviruses, influenza, respiratory infections, sexually transmitted infections, viruses, zoonoses, book review

The first edition of To Catch a Virus was published in 2013. Why is a second edition needed so soon (Figure)? Much has happened in virology over the past 10 years; the most notable occurrence was the COVID-19 pandemic, for which an entire new chapter was added. The title is a nod to the 1955 Hitchcock film To Catch a Thief*.* Rather than each chapter focusing on 1 virus or related family, the narrative is centered around discovery and diagnostics. Yellow fever is used to highlight the birth of virology and discovery of viruses as filterable agents. Polio, rabies, and influenza illustrate the use of animal models in research. Smallpox is used to elucidate the complex human immune system. 

**Figure:**
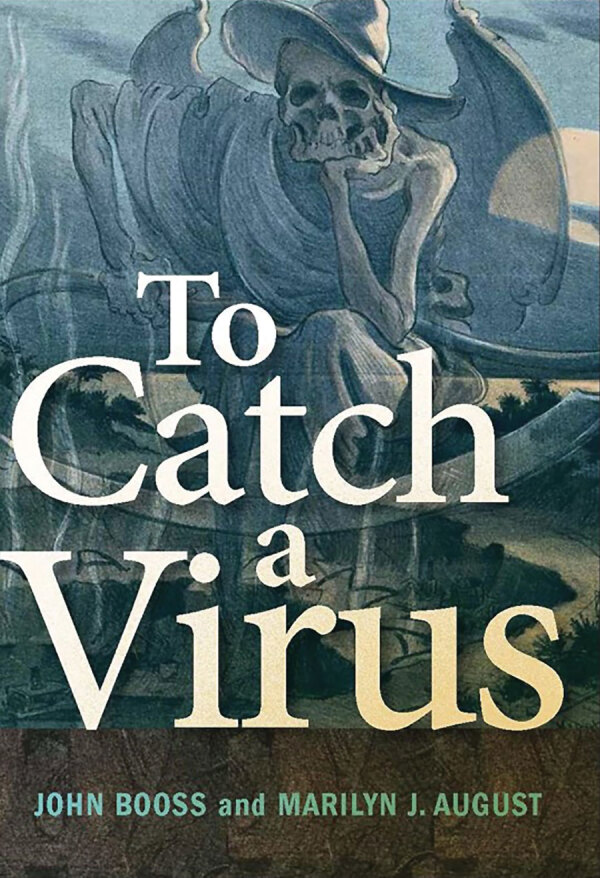
To Catch a Virus, 2nd Edition

Cellular pathology is shown for several viruses; reading about the move from light microscopy to electron microscopy is riveting. Discovery of the cytopathogenic effect was a turning point in virology, leading to diagnostic use of tissue culture assays; the momentous work on poliovirus is highlighted by tissue culture assay development. Many viruses were discovered starting in the 1950s. As coined by Robert J. Huebner, the “torrent of viral isolates” was the “virologist’s dilemma”: which isolates are associated with disease?

Those and other discoveries led to advances in diagnostics and patient management. In particular, HIV/AIDS research was transformative. Nucleic acid extraction, amplification, and measurements are now routine automated laboratory processes. Sequencing and bioinformatics methods are also now embedded in clinical and public health laboratories. The astonishing rapidity by which SARS-CoV-2 was discovered and then tracked by those techniques was breathtaking. Within 2 weeks of the first international notice of COVID-19 in December 2019, the entire SARS-CoV-2 genome sequence was posted online. Within 10 months, clinical trials evaluating the Pfizer-BioNTech mRNA vaccine showed 95% effectiveness. The story of the eventual discovery of the hepatitis B virus and routine detection by double diffusion in agar, electron microscopy, radioimmunoassay, and enzyme immunoassay/ELISA is fascinating to read.

Many historical figures are presented throughout the book. For example, the husband-and-wife team, Werner and Gertrude Henle, were productive scientists in virology, known for their development of the first influenza vaccine, but who also worked with mumps and Epstein-Barr viruses. At the start of the COVID-19 pandemic, Dr. Li Wenliang sent a warning to colleagues regarding what he had seen clinically before the government of China had acknowledged any cases. He was forced to sign a confession that declared he had made false statements. He returned to work at Wuhan Central Hospital, contracted COVID-19, and died on February 7, 2021. He was later exonerated.

The book is well referenced; the appendix has a useful timeline for each chapter. As early as 1957, Robert J. Huebner stated, “the virologist must be just as much an epidemiologist and clinician when studying the effect of prevalent nonfatal viruses in man as he is a well-grounded experimentalist or pathologist when studying similar effects in mice.” This book will appeal to virologists, microbiologists, clinicians, epidemiologists, other public health practitioners, and anyone who has an interest in the history of science or medicine. The book provides the history of virology, a dramatic story worth reading.

